# Pediatrics

**Published:** 1903-09

**Authors:** Maud J. Frye

**Affiliations:** Buffalo, N. Y.


### PROGRESS IN MEDICAL SCIENCE.

### Pediatrics.

CONDUCTED BY MAUD J. FRYE, M. D., Buffalo, N. Y.

### A NEW SIGN OF PLEURITIC EFFUSION IN CHILDREN.

KELLEY (*Archives of Pediatrics,* October, 1902,) calls attention to the well-known fact, that a child ill with pleurisy prefers to lie upon the afflicted side. With the accumulation of fluid he has observed that the child almost invariably prefers to turn and lie upon the back or to be propped up high in bed. He regards this instinctive changing of position as a sign of effusion, probably of considerable bulk. Sometimes following the emptying of the pleural cavity by aspiration the patient turns again upon the affected side to return to the dorsal decubitus if the fluid reaccumulates.

### ANTITOXIN IN DIPHTHERIA.

KERLEY (*Archives of Pediatrics,* October, 1902,) reports 159 cases of diphtheria, 103 treated without antitoxin with 60 deaths, and 56 with antitoxin with 4 deaths. He formulates the following rules for the giving of antitoxin:

With visible membrane inject at once and take a culture.

In croup, inject if there is inspiratory and expiratory obstruction.

The patients should be seen at 12 hour intervals.

Reinject in 12 hours if the patient is not improved, or if improvement is not marked. If continued improvement does not follow reinject at 12 hour intervals until the membrane disappears.

Dosage: 2,000 units for a child under 1 year of age, the amount to be repeated if necessary. Three thousand units for a child over 1 year of age, the amount to be repeated if necessary.

### ANEMIA OF CHILDHOOD.

HOLLOPETER (*Jour. Am. Med. Assn.,* January 31, 1903,) calls attention to certain frequently overlooked factors in inducing the anemias of childhood,—namely, primary dentition, dental decay, nasal stenosis and mouth breathing, and eye strain. He urges physicians to have in mind these causes in dealing with young patients.

### HEART DEPRESSION FOLLOWING ANTITOXIN.

HOLLADAY (*Virginia Medical Semi-monthly,* December 12, 1902,) reports a case of severe heart depression following the use of 500 c.c. of diphtheria antitoxin as an immunising dose. The patient was a male, aged 26 years. He became faint, cyanotic, pulseless at the wrist, the extremities being perfectly cold and covered with chilling perspiration. He complained of feeling as though a constricting band was forged around his chest. Under appropriate stimulation he slowly rallied. The physician had just administered to himself the same dose of antitoxin from the same bottle without untoward effect.

RETROPHARYNGEAL ABSCESS IN INFANCY.

Morse (*Jour. Am. Med. Assn.*, January 31, 1903,) discusses this condition. Retropharyngeal abscess is always preceded by retropharyngeal adentitis, the symptoms of which are very similar to nasopharyngeal catarrh. The fever is slight at first and inspection shows nothing definite. The enlarged nodes are easily felt. Suppuration when it occurs, usually develops in from 5 to 6 days. The abscess is in the lateral wall rather than in the back of the pharynx. With the advent of suppuration the temperature usually rises and becomes more irregular. There is unwillingness to take food or difficulty in swallowing. If the abscess is in the upper portion of the pharynx the cry is nasal and nasal respiration is chiefly disturbed. The mouth is kept open and the breathing is snoring and snuffling. If the abscess is low down the cry is laryngeal and the respiration is stridulous, like that in laryngeal spasm or stenosis. In these cases the respiratory murmur is diminished locally and many moist râles are heard. These may be due to a complicating bronchitis, but are usually the result of interference with respiration. In acute cases the head is held in a characteristic position, the neck extended, the head turned to one side. Swelling of the lymph nodes at the angle of the jaw may be very marked. In the majority of cases the tumor is visible but not infrequently, if low down, it cannot be seen. It can always be felt, although if low down the finger must be introduced deeply. The abscess is felt as a tense and elastic or a fluctuating tumor. If a simple adentitis be present the lymph nodes are felt as hard masses, round or oval in shape, and varying in size from a pea to that of a large bean. The examination of the throat is best made with a tongue depressor and not with a gag.

The treatment advised is poulticing to hasten suppuration. Open the abscess as soon as pus forms. The internal operation is preferred by Dr. Morse. The child is held in the upright position, being tipped forward the instant the abscess is opened. The abscess should be squeezed once or twice a day to keep up drainage and to prevent the opening from closing. Repeated incisions are sometimes necessary. Use an antiseptic mouth wash.

